# A Genome-Wide Association Study Confirms Previously Reported Loci for Type 2 Diabetes in Han Chinese

**DOI:** 10.1371/journal.pone.0022353

**Published:** 2011-07-22

**Authors:** Bin Cui, Xiaolin Zhu, Min Xu, Ting Guo, Dalong Zhu, Gang Chen, Xuejun Li, Lingyan Xu, Yufang Bi, Yuhong Chen, Yu Xu, Xiaoying Li, Weiqing Wang, Haifeng Wang, Wei Huang, Guang Ning

**Affiliations:** 1 Key Laboratory for Endocrine and Metabolic Diseases of Ministry of Health, Ruijin Hospital, Shanghai Jiao Tong University School of Medicine, Shanghai, China; 2 Department of Endocrine and Metabolic Diseases, Shanghai Clinical Center for Endocrine and Metabolic Diseases, Shanghai Institute of Endocrine and Metabolic Diseases, Ruijin Hospital, Shanghai Jiao Tong University School of Medicine, Shanghai, China; 3 Department of Endocrine Diseases, Nanjing Drum Tower Hospital, Affiliated Hospital of Nanjing University Medical School, Nanjing, China; 4 Department of Endocrine Diseases, Fujian Provincial Hospital, Fuzhou, China; 5 Department of Endocrine Diseases, First Affiliated Hospital of Xiamen University, Xiamen, China; 6 Department of Genetics, Chinese National Human Genome Center, Shanghai, China; University of Michigan, United States of America

## Abstract

**Background:**

Genome-wide association study (GWAS) has identified more than 30 loci associated with type 2 diabetes (T2D) in Caucasians. However, genomic understanding of T2D in Asians, especially Han Chinese, is still limited.

**Methods and Principal Findings:**

A two-stage GWAS was performed in Han Chinese from Mainland China. The discovery stage included 793 T2D cases and 806 healthy controls genotyped using Illumina Human 660- and 610-Quad BeadChips; and the replication stage included two independent case-control populations (a total of 4445 T2D cases and 4458 controls) genotyped using TaqMan assay. We validated the associations of KCNQ1 (rs163182, p = 2.085×10^−17^, OR 1.28) and C2CD4A/B (rs1370176, p = 3.677×10^−4^, OR 1.124; rs1436953, p = 7.753×10^−6^, OR 1.141; rs7172432, p = 4.001×10^−5^, OR 1.134) in Han Chinese.

**Conclusions and Significance:**

Our study represents the first GWAS of T2D with both discovery and replication sample sets recruited from Han Chinese men and women residing in Mainland China. We confirmed the associations of KCNQ1 and C2CD4A/B with T2D, with the latter for the first time being examined in Han Chinese. Arguably, eight more independent loci were replicated in our GWAS.

## Introduction

Type 2 diabetes (T2D) is a complex disease hallmarked by insulin resistance and pancreatic beta-cell dysfunction [1]. T2D is becoming a major concern of global public health due to its escalating prevalence throughout the world [2]. In China, 9.7% and 15.5% of the entire population suffer from T2D and prediabetes, respectively [3]. Although overfeeding and sedentary lifestyle are claimed as the main contributors to its increasing incidence, genetic factors play a significant role in the etiology and pathogenesis of T2D, in many cases via interaction with environmental counterparts [4].

Genetic analysis of T2D and related diseases (such as obesity and monogenic diabetes) and traits (such as fasting plasma glucose levels) has greatly improved our understanding of glucose homeostasis and energy balance in both physiological and pathological conditions, some of which has brought novel preventive and therapeutic options [4]. Before the year 2007, studies based on linkage analysis and candidate gene strategy identified only a few genetic loci of T2D [5]–[7]. More recently, several genome-wide association studies (GWASs) have been completed in independent population samples derived from Caucasians and Japanese, and identified a host of novel susceptibility loci of T2D [8]–[19]. With a more recent large-scale meta-analysis taking into account [20], these studies in total have discovered at least 38 independent susceptibility loci of T2D, many of which have been replicated in populations of different ancestries, including Han Chinese [21], [22].

Extending GWAS to populations of diverse descents is valuable, because different frequencies of genetic variants and patterns of linkage disequilibrium (LD) due to population backgrounds may strongly affect the power and potential of GWAS to discover and/or refine certain genetic loci associated with disease [23]. For example, association of *KCNQ1* with T2D was first independently identified by two GWASs in Japanese [14], [15], although for both studies the sample size of the discovery stage was small and the genomic coverage of SNPs insufficiently dense [24]. However, to date, original data of GWAS of T2D in Han Chinese is still limited [25], [26]. One study was conducted among Han Chinese in Taiwan that identified two additional novel loci in the protein tyrosine phosphatase receptor type D (*PTPRD*) and serine racemase (*SRR*) genes [25]. The other was performed in women from Shanghai Women's Health Study (SWHS) and Shanghai Breast Cancer Study (SBCS) [26]. Here, we reported a 2-stage study, comprising one discovery and one replication stage, in both Han Chinese men and women residing in Mainland China.

## Results

In the discovery stage, 998 T2D patients of Han Chinese descent recruited in Shanghai were genotyped using Illumina Human 660-Quad BeadChips. Our control population was derived from a glucose survey in a community in Shanghai and was genotyped using Illumina Human 660- and 610-Quad BeadChips. After stringent quality control, we obtained 561,694 SNPs that were common to both genotyping platforms with both average call rates of >99%. To ensure that case and control groups were genetically matched, in addition to close examination of their geographic origins, MDS was used to exclude population outliers, the result of which was further confirmed by PCA, which showed minimal evidence for population stratification [Figure S1, Figure S2]. 474,515 SNPs in 793 cases and 806 controls entered final statistical analysis using the Cochran-Armitage trend test to examine the genotype-phenotype association under an additive model. After genomic control (GC) with an inflation factor λ of 1.08 [Figure S3], the association results did not change significantly.

**Figure 1:**
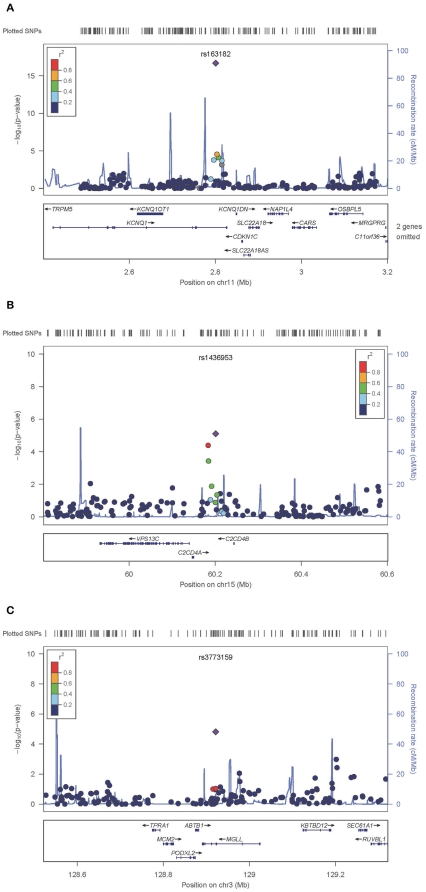
Association signals at three chromosome regions. Regional plots displays association results for SNPs as a function of genomic distance (chromosomal position from National Center for Biotechnology Information build hg18) for *KCNQ1*(Panel A), *C2CD4A/B* (Panel B), and *MGLL* (Panel C). Top line represents genomic coverage at each locus; with each vertical tick representing genotyped SNPs. Purple diamonds indicate SNP at each locus with the strongest association. Each circle represents a SNP, with the color of the circle indicating the correlation between that SNP and the best SNP at the locus (purple diamond). Light blue lines indicate estimated recombination hot spots in HapMap. Bottom panel shows genes at each locus as annotated in the UCSC Genome Browser Annotation Database.

We selected the top 30 significantly associated SNPs representing 15 genomic regions in the discovery stage (arbitrarily defined as trend-*P* ≤10^−4^) for genotyping in an independent case-control population from Shanghai (n = 2620) (1058 cases and 1562 controls, part of Replication 1) as a fast-track replication analysis [Table S1, Figure S4]. Among the 30 SNPs, 3 representing 2 genomic loci were replicated with the same direction of association with the discovery stage at the significance level of *P*<0.10 (Figure 1). Two of them, namely rs163182 and rs163184 (*P* = 2.348×10^−7^, OR = 1.37, and *P* = 0.04332, OR = 1.121, respectively), were located in the same locus in gene *KCNQ1* as previously reported. The remaining SNP, rs3773159 (*P* = 0.09591, OR = 1.152), is likely to represent a novel susceptibility locus for T2D in the population.

We genotyped the rest of replication 1 (1602 cases and 506 controls residing in Shanghai and Jiangsu Province) and pooled analysis (Replication 1: 2660 cases and 2068 controls) showed a nominally significant association of rs3773159 with T2D (*P* = 0.0418, OR = 1.137, Table 1). Further genotyping and analysis in an independent sample comprising 1785 cases and 2390 controls (Replication 2: Southern Han) identified a consistent association though without reaching significance level. Therefore, there was not enough evidence to establish the association of rs3773159 with T2D in out populations.

**Table 1:** SNPs associated with T2D in the Han Chinese population.

	risk allele	sample size (T2D/NC)	genotyping rate	RAF in T2D	RAF in NC	OR 95% confidence interval	trend-Punadjusted
***KCNQ1***							
rs163182							
GWAS	C	793/806		0.402	0.332	1.353	4.51×10^−5^
Central Han	C	2660/2068	98.35%	0.387	0.326	1.305 (1.198–1.423)	3.543×10^−9^
Southern Han	C	1785/2390	99.04%	0.411	0.362	1.227 (1.122–1.342)	1.205×10^−5^
Combined	C	5238/5264		0.397	0.343	1.280	2.085×10^−17^
***C2CD4A/B***							
rs1370176							
GWAS	C	793/806		0.731	0.687	1.238	0.005546
Central Han	C	2158/1562	99.14%	0.720	0.698	1.115 (1.007**-**1.234)	0.03597
Southern Han	C	1785/2390	98.37%	0.739	0.722	1.088 (0.986**-**1.201)	0.09241
Combined	C	4736/4758		0.729	0.708	1.124	3.677×10^−4^
rs1436953							
GWAS	G	793/806		0.668	0.617	1.246	0.002698
Central Han	G	2660/2068	98.73%	0.641	0.601	1.187 (1.091–1.292)	9.671×10^−5^
Southern Han	G	1785/2390	99.14%	0.698	0.688	1.045 (0.950–1.148)	0.3535
Combined	G	5238/5264		0.664	0.643	1.141	7.753×10^−6^
rs7172432							
GWAS	A	793/806		0.644	0.582	1.302	3.264×10^−4^
Central Han	A	2158/1562	98.76%	0.630	0.589	1.187 (1.080–1.306)	4.529×10^−4^
Southern Han	A	1785/2390	98.42%	0.666	0.661	1.025 (0.993–1.058)	0.6154
Combined	A	4736/4758		0.646	0.624	1.134	4.001×10^−5^
***MGLL***							
rs3773159							
GWAS	T	793/806		0.183	0.107	1.879	2.64×10^−7^
Central Han	T	2660/2068	97.95%	0.134	0.120	1.137 (1.004–1.287)	0.0418
Southern Han	T	1785/2390	99.33%	0.141	0.134	1.061 (0.9354–1.204)	0.3507
Combined	T	5238/5264		0.144	0.124	1.196	1.552×10^−5^

RAF(T2D) and RAF(NC), risk allele frequency in T2D cases and controls, respectively. OR, odds ratio for risk allele.

We checked a total of 37 genomic loci previously reported to be associated with T2D [20] in our discovery stage data, among which 42 SNPs in 26 loci were successfully genotyped. 13 SNPs representing 9 loci were significantly associated with T2D in our GWAS at a significant level of *P*<0.05 (Table S2): rs2793831 (proxy for rs10923931, *NOTCH2*), rs2943641 (*IRS1*), rs2120825 (proxy for rs1801282, *PPARG*), rs734312 (*WFS1*), rs896854 (*TP53INP1*), rs10906115 (*CDC123/CAMK1D*), rs7901695 (*TCF7L2*), rs7903146 (*TCF7L2*), rs1552224 (*CENTD2*), rs1370176 (*C2CD4A/B*), rs1436953 (*C2CD4A/B*), rs7172432 (*C2CD4A/B*), and rs1436955 (*C2CD4B*). If we adopt a less stringent threshold of *P*<0.10, 4 more SNPs representing 3 genomic regions showed significant association with T2D in the discovery stage (Table S2): rs1111875 (*HHEX*), rs7923837 (*HHEX*), rs8050136 (*FTO*), and rs780094 (*GCKR*).

We noticed that the three SNPs – rs1370176, rs1436953, and rs7172432 in *C2CD4A/B* on chromosome 15 – were exactly the lead SNPs in a three-stage GWAS recently reported involving a total of ∼︀19000 Japanese [27]. Further genotyping in the fast-track replication sample showed that the three SNPs tended to be consistently associated with T2D (*P* = 0.1134, 0.03651, and 0.01884, respectively). Genotyping in the remaining 1602 cases and 506 controls of Replication 1 and pooled analysis (Replication 1) expectedly rendered these associations significant (rs1370176, *P* = 0.03597, OR = 1.115; rs1436953, *P* = 9.671×10^−5^, OR = 1.187; rs7172432, *P* = 4.529×10^−4^, OR = 1.187, respectively; Table 1). Further analysis in the Southern Han population (Replication 2) showed that the associations were in the same direction as those in Central Han population but did not reach significance level (Table 1, Figure 1).

## Discussion

Our GWAS did not provide sufficient evidence for potentially novel genetic variation associated with T2D in Han Chinese. However, our study validated 10 previously reported loci associated with T2D, including *KCNQ1* and *C2CD4A/B*, and several of which were very recently discovered by large-scale multistage study and meta-analyses.

In Han Chinese population, our study validated previously known susceptibility loci of T2D, among which SNPs in *KCNQ1*were consistently and most strongly associated with T2D (rs163182, *P_combined_* = 2.085×10^−17^, OR = 1.280). rs163184, the SNP most significantly associated with T2D in *KCNQ1* in our discovery stage, is also the lead SNP in two other independent studies [14], [20]. One additional SNP in the same locus, namely rs2237892, was reported to be significantly associated with T2D in two independent studies in Japanese [14], [15], and replicated in our previous study [22]. Collectively, these data confirmed that variants in *KCNQ1* were associated with T2D in different populations, and our GWAS worked in identifying such variants.

Aside from variants in *KCNQ1*, our study rediscovered several SNPs in additional susceptibility loci of T2D originally identified in populations of different ancestries, and SNPs in three such loci came to our notice. The first is *C2CD4A/B* in 15q21.3, which was quite recently reported by a GWAS of T2D involving about 8000 Japanese in their discovery stage [27]. The most significant SNP reported, rs7172432, was also most strongly associated with T2D at the locus in the Han Chinese population (*P_GWAS_* = 3.264×10^−4^), but did not reach the cutoff *P* = 10^−4^, mainly due to our limited power because of small sample size. Two more SNPs reported, namely rs1370176 and rs1436953, were in the same locus and associated with T2D in our GWAS. We noticed that another recent work reported an association of rs1436955 with T2D in Han Chinese, which was indeed in the same locus; albeit *in silico* replication strategy might have prevented their further analysis [26]. Although not replicated in Southern Han population possibly due to population reasons, the 3 SNPs were significantly associated with T2D in Central Han population, with risk alleles having slightly stronger effect sizes (OR = 1.115–1.187) than those in Japanese population as previously reported [27]. These results present direct evidence that genetic variants in *C2CD4A/B* locus are associated with T2D in Central Han Chinese population residing in Mainland China. Further studies are required to investigate whether such associations exist in Southern and Northern Han Chinese populations and identify the causal variants.

The other two loci are *TP53INP1* (rs896854, *P_GWAS_* = 0.002212) and *CENTD2* (rs1552224, *P_GWAS_* = 0.04226); both of them were discovered in a large-scale meta-analysis comprising more than one hundred thousand individuals of European descent (DIAGRAM+). Our GWAS was likely to validate these two newly identified loci in the Han Chinese population, but further replication is required to examine their effects. Moreover, in light of the small sample size of our GWAS stage, these results support the notion that GWAS in Han Chinese has potential to identify novel risk loci of T2D.

Our study represents the first GWAS with both discovery and replication sample sets recruited both Han Chinese men and women residing in Mainland China. Han Chinese is geographically and genetically heterogeneous and has subpopulation structures, which may have considerable effect on design and interpretation of GWAS [28]. Allele effects in Southern Han Chinese were consistently weaker than those in Central Han Chinese (Table 1); we consider this as a result of different population backgrounds. Because T2D-associated variants show much weaker effects than alleles associated with other diseases and traits (e.g., autoimmune disease and malignancies), a much larger sample consisting of homogenous individuals is required for genuine associations to achieve genome-wide significance. Though our initial sample size is small, which might have prevented us to discover more potentially novel associations, our GWAS has successfully replicated 10 previously reported T2D susceptibility loci, several of which are very recently discovered by large-scale studies and meta-analyses in Caucasians and Japanese. This fact lends convincing evidence of the soundness of our study, and highlights the potential of discovering novel genetic variations associated with T2D by extending GWAS to diverse populations.

In conclusion, our genome-wide association study confirmed several T2D susceptibility loci previously identified in Caucasians and Japanese, among which variants in *KCNQ1*showed the strongest association, and variants in *C2CD4A/B* were first replicated in Han Chinese residing in Mainland China.

## Methods

### Ethics statement

This study was approved by the Institutional Review Board of the Ruijin Hospital, Shanghai Jiao Tong University School of Medicine and was in accordance with the principle of the Helsinki Declaration II. The written informed consent was obtained from each participant.

### Study populations

The GWAS genotyped 998 T2D patients recruited from outpatient departments of Ruijin Hospital, and 1002 healthy controls obtained from one glucose survey in Youyi community, Baoshan district, Shanghai [29]. The replication analysis included two independent populations (Replication 1: 2660 T2D cases and 2068 controls residing in Shanghai [22] and Jiangsu province [Central Han]; Replication 2: 1785 T2D cases and 2390 controls residing in Southern China [Southern Han]) [28]. All participants self-reported as Han Chinese. T2D case was diagnosed according to the 1999 World Health Organization criteria (fasting plasma glucose level ≥7.0 mmol/l and/or 2h oral glucose tolerance test (OGTT) plasma glucose level ≥11.1 mmol/l) or with taking anti-diabetic therapies. The controls were defined as a fasting glucose level less than 6.1 mmol/l and a 2 h OGTT plasma glucose level less than 7.8 mmol/l.

### Genotyping and quality control

Genomic DNA was extracted from peripheral blood by standard phenol/chloroform-based method. In the discovery stage, genotyping was conducted by using Illumina Human 660- and 610-Quad BeadChips at the Chinese National Human Genome Center at Shanghai. Genotyping was performed according to the Infinium HD protocol from Illumina. Quality control involved exclusion of SNPs with a call rate <90%, a minimum allele frequency <0.01, and/or a significant deviation from Hardy-Weinberg equilibrium (HWE) in the controls (*P*<10^−7^). SNPs on the X, Y and mitochondrial chromosomes and copy number variation (CNV) probes were also excluded from further analysis.

In the replication stage, genotyping was conducted using 5′ nuclease allelic discrimination assay (TaqMan Assay) on an ABI PRISM 7900HT Sequence Detection System following the manufacturer's protocol. In our study, the call rate ranged from 97.95% (rs3773159 in the Replication 1) to 99.33% (rs3773159 in Replication 2). There is no significant difference of SNP calling between the case and the control groups. The average consensus rate in the duplicate samples (n = 100) was 100%.

### Statistical analysis

Identification of cryptic relatedness among individuals in the discovery stage was based on pairwise identity by state using the PLINK 1.07 software [30], after which one of the two related individuals was excluded. Population structure of the remaining sample was examined and outliers were excluded based on multidimensional scaling analysis (MDS) using PLINK 1.07 as well as principal component analysis (PCA) using EIGENSTRAT software [31]. Cochran-Armitage trend test was used to examine the association of genotype with disease phenotype and calculate odds ratio (OR) per allele. The quantile-quantile plot was employed to evaluate the overall significance of the genome-wide association results and impacts of population stratification. The genomic control inflation factor λwas also calculated to examine the effects of population stratification.

Replication analysis was performed by first analyzing replication samples separately and then combining them with the discovery sample set [32]. Association analysis of the combined samples was performed based on Cochran-Mantel-Haenszel tests [33]. Joint analysis was performed using PLINK under a fixed-effect model [30].

## Supporting Information

Figure S1 **Multidimensional scaling analysis (MDS) plot.** MDS plot by PLINK of the 793 cases and 806 controls shows no evident population stratification and outliers. Blue: control; pink: case.
(TIF)

Figure S2 **Principal component analysis (PCA) plot.** PCA plot by EIGENSTRAT of the 793 cases and 806 controls shows no evident population stratification and outliers. Green: control; red: case.
(TIF)

Figure S3 **Quantile-quantile (Q-Q) plot for the trend test. (λ = 1.08).** Q-Q plot for the Cochran-Armitage trend test for 474,515 SNPs in 793 cases and 806 controls. λ = 1.08 and minimal evidence of association due to population stratification was observed.
(TIF)

Figure S4 **Manhattan plot for GWAS data.** The x-axis represents chromosomal location of 474,515 SNPs examined and the y-axis represents –log10 of the P value of the Cochran-Armitage trend test under an additive model. A cutoff line was drawn at the significance threshold of 10^−4^.
(TIF)

Table S1 **SNPs selected for fast-track replication.** RAF(T2D) and RAF(NC), risk allele frequency in T2D cases and controls, respectively. OR, odds ratio for risk allele.
(DOC)

Table S2 **SNPs in previously reported T2D loci.** RAF(T2D) and RAF(NC), risk allele frequency in T2D cases and controls, respectively. OR, odds ratio for risk allele. *, P<0.05. **, P<0.10
(DOC)
