# Supplementary figures and images for: A Genome-Wide Association Study Confirms Previously Reported Loci for Type 2 Diabetes in Han Chinese
Source: PLoS One. 2011 Jul 22;6(7):e22353. doi: 10.1371/journal.pone.0022353 (PMC3142153; doi:10.1371/journal.pone.0022353)

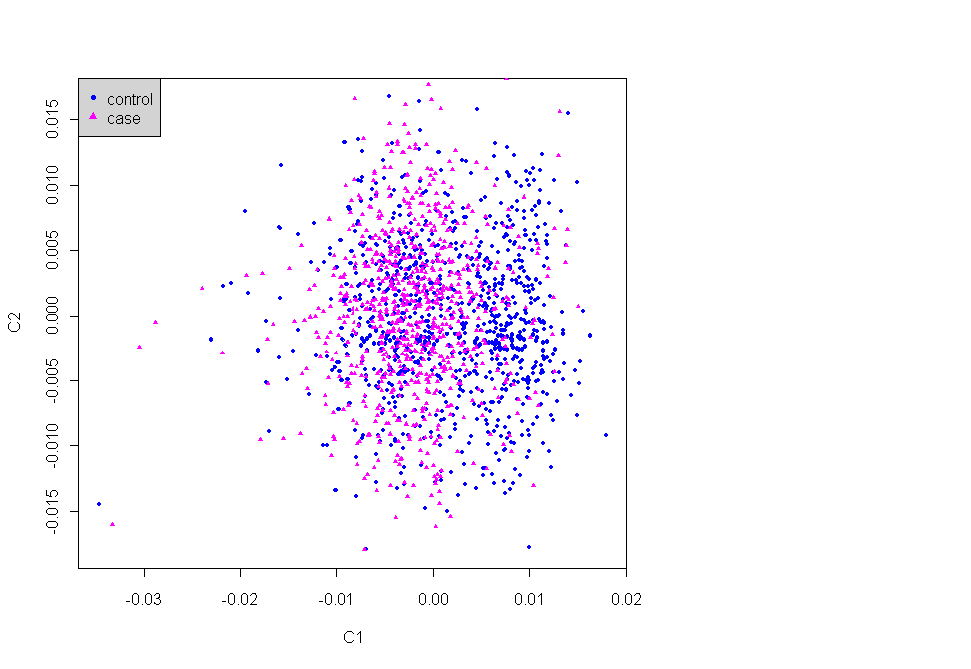

Supplement: Figure S1 — Multidimensional scaling analysis (MDS) plot. MDS plot by PLINK of the 793 cases and 806 controls shows no evident population stratification and outliers. Blue: control; pink: case. (TIF) [file pone.0022353.s001.tif]

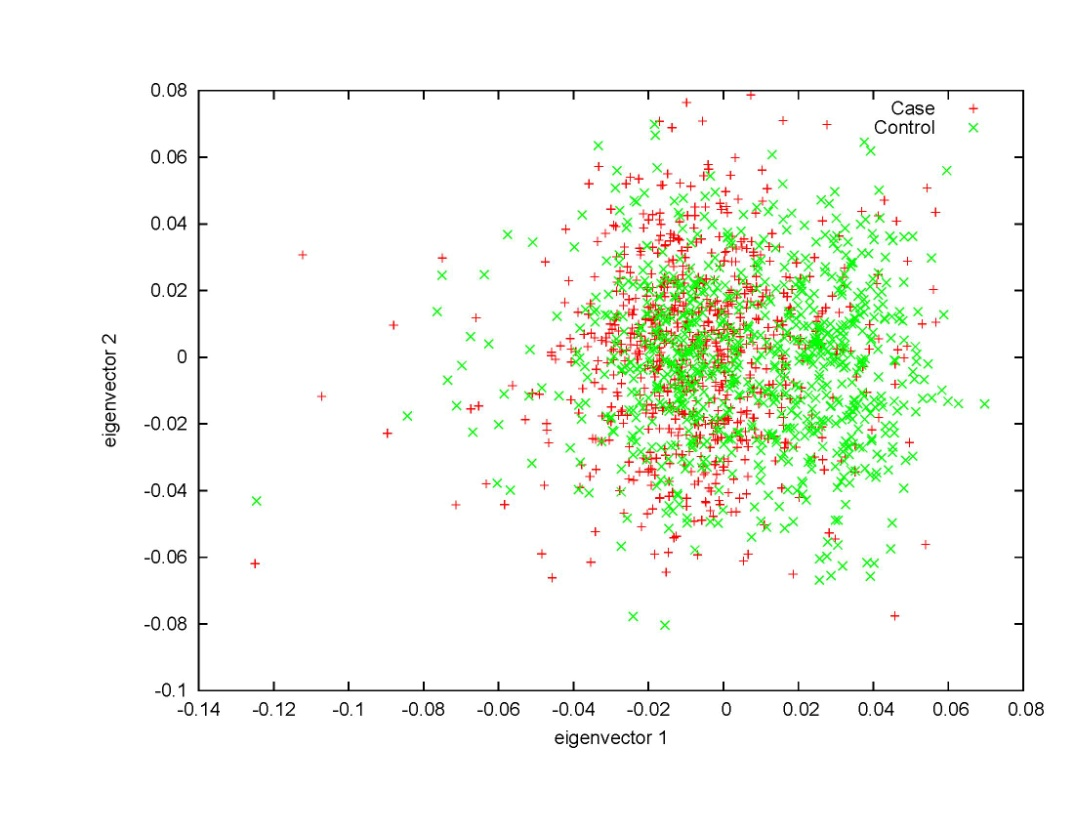

Supplement: Figure S2 — Principal component analysis (PCA) plot. PCA plot by EIGENSTRAT of the 793 cases and 806 controls shows no evident population stratification and outliers. Green: control; red: case. (TIF) [file pone.0022353.s002.tif]

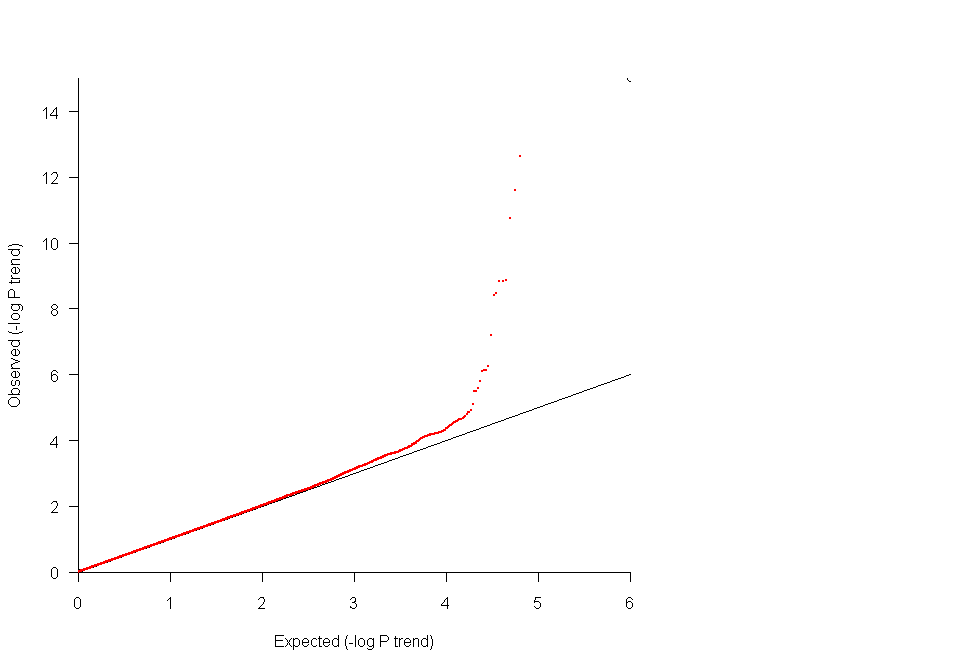

Supplement: Figure S3 — Quantile-quantile (Q-Q) plot for the trend test. (λ = 1.08). Q-Q plot for the Cochran-Armitage trend test for 474,515 SNPs in 793 cases and 806 controls. λ = 1.08 and minimal evidence of association due to population stratification was observed. (TIF) [file pone.0022353.s003.tif]

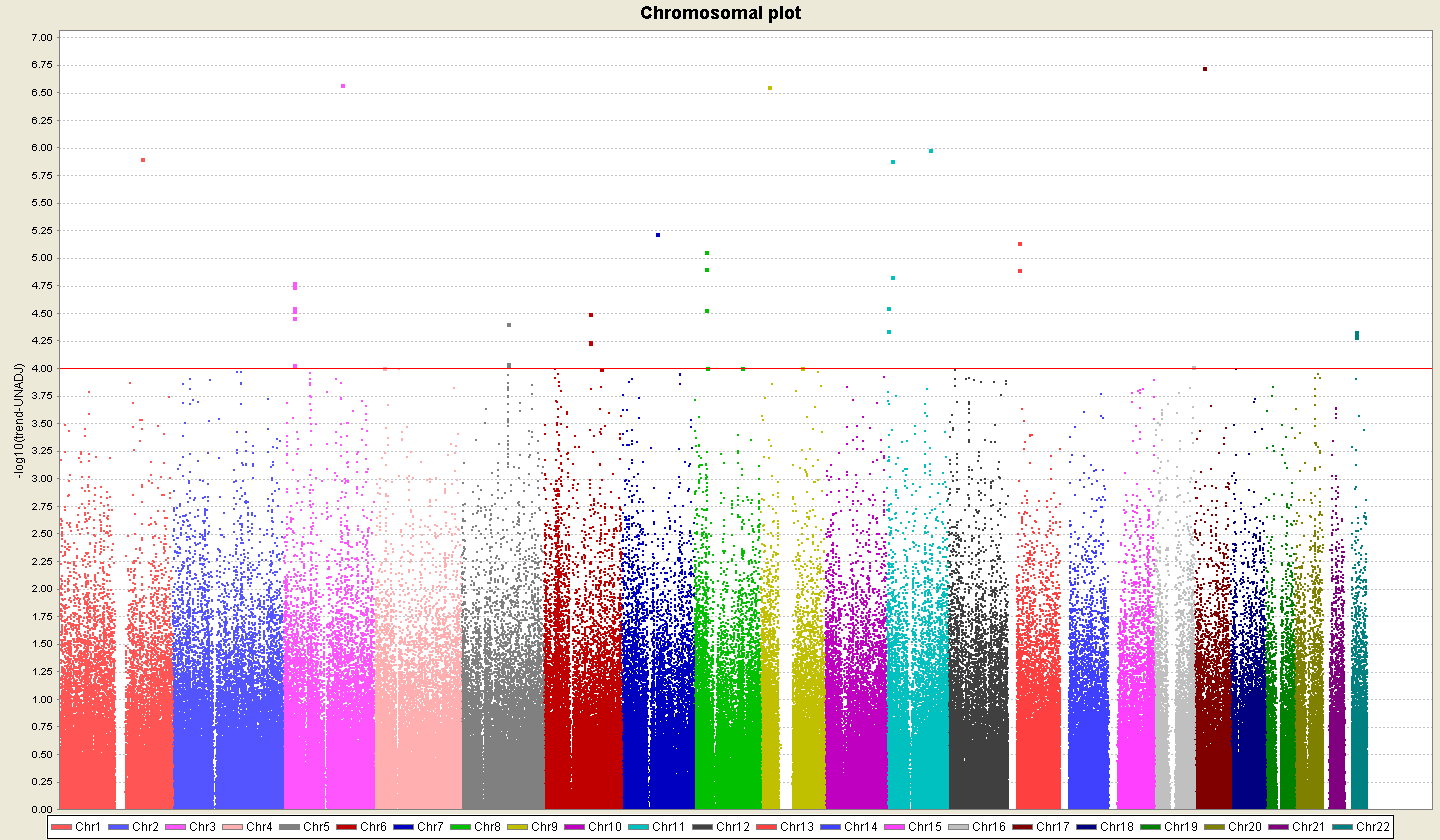

Supplement: Figure S4 — Manhattan plot for GWAS data. The x-axis represents chromosomal location of 474,515 SNPs examined and the y-axis represents –log10 of the P value of the Cochran-Armitage trend test under an additive model. A cutoff line was drawn at the significance threshold of 10−4. (TIF) [file pone.0022353.s004.tif]
