# Supplementary material for: A Genome-Wide Association Study Confirms Previously Reported Loci for Type 2 Diabetes in Han Chinese
Source: PLoS One. 2011 Jul 22;6(7):e22353. doi: 10.1371/journal.pone.0022353 (PMC3142153; doi:10.1371/journal.pone.0022353)
Supplement: Table S1 — SNPs selected for fast-track replication. RAF(T2D) and RAF(NC), risk allele frequency in T2D cases and controls, respectively. OR, odds ratio for risk allele. (DOC) [file pone.0022353.s005.doc]

**Table S1.** SNPs selected for fast-track replication.

|  |  |  |  |  | GWAS | | | | Fast-track Replication (1058 cases + 1562 controls) | | | |
| --- | --- | --- | --- | --- | --- | --- | --- | --- | --- | --- | --- | --- |
|  | dbSNP ID | Chr. | Nearest  Gene(s) | Risk  allele | RAF  (T2D) | RAF  (NC) | OR | *P* value (trend) | RAF  (T2D) | RAF  (NC) | OR | *P valu*e (trend) |
| 1 | rs4517314 | 1 | *CACNA1E* | C | 0.82 | 0.88 | 0.62 | 1.25×10^-6^ | 0.84 | 0.83 | 1.07 | 0.6045 |
| 2 | rs7612158 | 3 | *THRB* | G | 0.85 | 0.90 | 0.63 | 1.65×10^-5^ | 0.88 | 0.87 | 1.18 | 0.03317 |
| 3 | rs6781758 | 3 | *THRB* | G | 0.85 | 0.90 | 0.65 | 9.10×10^-5^ | 0.88 | 0.87 | 1.12 | 0.1877 |
| 4 | rs2360959 | 3 | *THRB* | G | 0.85 | 0.90 | 0.64 | 2.97×10^-5^ | 0.88 | 0.86 | 1.23 | 0.009088 |
| 5 | rs2360958 | 3 | *THRB* | A | 0.84 | 0.89 | 0.64 | 1.79×10^-5^ | 0.88 | 0.86 | 1.16 | 0.1309 |
| 6 | rs1511540 | 3 | *THRB* | G | 0.85 | 0.91 | 0.64 | 3.44×10^-5^ | 0.88 | 0.86 | 1.19 | 0.02064 |
| 7 | rs6782978 | 3 | *THRB* | G | 0.88 | 0.93 | 0.59 | 2.78×10^-5^ | 0.91 | 0.90 | 1.07 | 0.5191 |
| 8 | rs3773159 | 3 | *MGLL* | T | 0.18 | 0.11 | 1.88 | 2.64×10^-7^ | 0.13 | 0.12 | 1.15 | 0.09591 |
| 9 | rs896545 | 5 | *SLCO6A1* | G | 0.47 | 0.54 | 0.75 | 9.48×10^-5^ | 0.52 | 0.52 | 1.03 | 0.4999 |
| 10 | rs2400796 | 5 | *SLCO6A1* | C | 0.47 | 0.54 | 0.75 | 9.03×10^-5^ | 0.51 | 0.51 | 1.02 | 0.69 |
| 11 | rs953280 | 5 | *SLCO6A1* | C | 0.39 | 0.32 | 1.36 | 3.93×10^-5^ | 0.35 | 0.34 | 1.04 | 0.5179 |
| 12 | rs6570772 | 6 | *SIM1/ASCC3* | T | 0.13 | 0.18 | 0.66 | 3.15×10^-5^ | 0.16 | 0.14 | 1.13 | 0.1395 |
| 13 | rs7751526 | 6 | *SIM1/ASCC3* | A | 0.19 | 0.24 | 0.70 | 5.76×10^-5^ | 0.23 | 0.22 | 1.04 | 0.5205 |
| 14 | rs240766 | 6 | *SIM1/ASCC3* | G | 0.22 | 0.29 | 0.72 | 5.72×10^-5^ | 0.28 | 0.25 | 1.13 | 0.05738 |
| 15 | rs16885926 | 7 | *MAGI2* | G | 0.68 | 0.76 | 0.68 | 5.99×10^-6^ | 0.75 | 0.73 | 1.14 | 0.06269 |
| 16 | rs2666172 | 8 | *DOCK5* | C | 0.21 | 0.27 | 0.71 | 2.91×10^-5^ | 0.25 | 0.22 | 1.19 | 0.008565 |
| 17 | rs2468896 | 8 | *DOCK5* | G | 0.23 | 0.30 | 0.70 | 8.70×10^-6^ | 0.26 | 0.25 | 1.07 | 0.2883 |
| 18 | rs7012335 | 8 | *DOCK5* | G | 0.23 | 0.30 | 0.70 | 1.24×10^-5^ | 0.28 | 0.25 | 1.14 | 0.04449 |
| 19 | rs2779772 | 9 | *CNTLN* | A | 0.42 | 0.52 | 0.68 | 2.79×10^-7^ | 0.49 | 0.47 | 1.10 | 0.1674 |
| 20 | rs163182 | 11 | *KCNQ1* | C | 0.40 | 0.33 | 1.35 | 4.51×10^-5^ | 0.40 | 0.32 | 1.37 | 2.348×10^-7^ |
| 21 | rs163184 | 11 | *KCNQ1* | C | 0.49 | 0.41 | 1.35 | 2.82×10^-5^ | 0.47 | 0.44 | 1.12 | 0.04332 |
| 22 | rs4375432 | 11 | *GALNTL4* | T | 0.51 | 0.43 | 1.42 | 1.29×10^-6^ | 0.51 | 0.49 | 1.09 | 0.2283 |
| 23 | rs4910306 | 11 | *GALNTL4* | G | 0.43 | 0.51 | 0.73 | 1.45×10^-5^ | 0.46 | 0.45 | 1.02 | 0.6602 |
| 24 | rs559477 | 11 | *MAML2* | C | 0.39 | 0.49 | 0.67 | 1.03×10^-6^ | 0.54 | 0.50 | 1.16 | 0.08674 |
| 25 | rs5021764 | 13 |  | C | 0.62 | 0.55 | 1.38 | 7.18×10^-6^ | 0.60 | 0.58 | 1.06 | 0.2988 |
| 26 | rs4770557 | 13 |  | A | 0.62 | 0.55 | 1.37 | 1.26×10^-5^ | 0.59 | 0.58 | 1.03 | 0.5555 |
| 27 | rs7208434 | 17 |  | G | 0.74 | 0.66 | 1.48 | 1.87×10^-7^ | 0.74 | 0.72 | 1.05 | 0.4347 |
| 28 | rs2071862 | 22 | *TPST2/MIAT* | G | 0.57 | 0.64 | 0.75 | 5.14×10^-5^ | 0.58 | 0.57 | 1.07 | 0.3052 |
| 29 | rs6005101 | 22 | *TPST2/MIAT* | C | 0.48 | 0.55 | 0.75 | 4.83×10^-5^ | 0.51 | 0.50 | 1.03 | 0.6338 |
| 30 | rs739312 | 22 | *MIAT* | A | 0.57 | 0.64 | 0.74 | 4.60×10^-5^ | 0.61 | 0.60 | 1.03 | 0.5836 |
|  |  |  |  |  |  |  |  |  |  |  |  |  |
