# Supplementary material for: A Genome-Wide Association Study Confirms Previously Reported Loci for Type 2 Diabetes in Han Chinese
Source: PLoS One. 2011 Jul 22;6(7):e22353. doi: 10.1371/journal.pone.0022353 (PMC3142153; doi:10.1371/journal.pone.0022353)
Supplement: Table S2 — SNPs in previously reported T2D loci. RAF(T2D) and RAF(NC), risk allele frequency in T2D cases and controls, respectively. OR, odds ratio for risk allele. *, P<0.05. **, P<0.10 (DOC) [file pone.0022353.s006.doc]

**Table S2.** SNPs in loci previously reported to be associated with T2D.

|  |  |  |  | This Study (Stage 1) | | | Our previous replication study [22] |
| --- | --- | --- | --- | --- | --- | --- | --- |
| Our Genes | Chr | Reported  SNP | Examined  SNP | RAF in T2D | RAF in NC | *P* (trend) | *P* value (additive) |
| *NOTCH2* | 1 | rs10923931 | rs2793831 | 0.978 | 0.963 **0.01568*** | **0.01568*** |  |
| *PROX1* | 1 | rs340874 | rs340874 | 0.391 | 0.382 | 0.6076 |  |
| *IRS1* | 2 | rs2493641 | rs2493641 | 0.946 | 0.926 **0.02623*** | **0.02623*** |  |
|  |  | rs7578326 | rs7578326 | 0.141 | 0.135 | 0.5712 |  |
| *GCKR* | 2 | rs780094 | rs780094 | 0.484 | 0.452 **0.07547**** | **0.07547**** |  |
| *BCL11A* | 2 | rs243021 | rs243021 | 0.322 | 0.308 | 0.4009 |  |
| *PPARG* | 3 | rs1801282 | rs2120825 | 0.953 | 0.937 **0.04969*** | **0.04969*** | 0.18 |
| *IGF2BP2* | 3 | rs4402960 | rs4402960 | 0.258 | 0.243 | 0.3146 | **0.01** |
|  |  | rs1470579 | rs1470579 | 0.275 | 0.256 | 0.2211 | **0.03** |
| *ADAMTS9* | 3 | rs4607103 | rs7638389 | 0.363 | 0.358 | 0.7559 |  |
|  |  | rs6795735 | rs6795735 | 0.755 | 0.749 | 0.7282 |  |
| *WFS1* | 4 | rs734312 | rs734312 | 0.849 | 0.818 **0.01973*** | **0.01973*** |  |
| *CDKAL1* | 6 | rs7754840 | rs7754840 | 0.444 | 0.427 | 0.3176 |  |
|  |  | rs9295475 | rs9295475 | 0.444 | 0.426 | 0.3006 |  |
|  |  | rs7756992 | rs7756992 | 0.542 | 0.536 | 0.7251 | **0.0001** |
|  |  | rs4134943 | rs4134943 | 0.931 | 0.930 | 0.8753 |  |
| *JAZF1* | 7 | rs864745 | rs917117 | 0.243 | 0.238 | 0.6968 |  |
|  |  | rs849139 | rs849139 | 0.480 | 0.464 | 0.3766 |  |
| *GCK* | 7 | rs730497 | rs730497 | 0.233 | 0.220 | 0.3785 |  |
| *DGKB/TMEM195* | 7 | rs2191349 | rs2191348 | 0.651 | 0.650 | 0.9824 |  |
| *SLC30A8* | 8 | rs13266634 | rs13266634 | 0.584 | 0.575 | 0.6059 | **0.0002** |
| *TP53INP1* | 8 | rs896854 | rs896854 | 0.335 | 0.285 **0.002212*** | **0.002212*** |  |
| *PTPRD* | 9 | rs17584499 | rs17584499 | 0.094 | 0.081 | 0.1646 |  |
| *TCF7L2* | 10 | rs7903146 | rs7903146 | 0.048 | 0.032 **0.01887*** | **0.01887*** |  |
|  |  | rs7901695 | rs7901695 | 0.048 | 0.032 **0.01887*** | **0.01887*** |  |
| *HHEX* | 10 | rs5015480 | rs5015480 | 0.176 | 0.166 | 0.4360 | 0.30 |
|  |  | rs1111875 | rs1111875 | 0.293 | 0.266 **0.08378**** | **0.08378**** | **0.08** |
|  |  | rs7923837 | rs7923837 | 0.213 | 0.190 **0.09938**** | **0.09938**** | 0.21 |
| *CDC123/CAMK1D* | 10 | rs10906115 | rs10906115 | 0.646 | 0.610 **0.03871*** | **0.03934*** |  |
| *KCNJ11* | 11 | rs5219 | rs5219 | 0.604 | 0.588 | 0.3538 |  |
|  | 11 | rs5215 | rs5215 | 0.604 | 0.589 | 0.3761 | **0.03** |
| *MTNR1B* | 11 | rs1387153 | rs1387153 | 0.421 | 0.401 | 0.2720 | 0.63 |
| *CENTD2* | 11 | rs1552224 | rs1552224 | 0.927 | 0.907 **0.04226*** | **0.04226*** |  |
| *C2CD4A/B* | 15 | rs7172432 | rs7172432 | 0.644 | 0.582 **0.0003264*** | **0.0003264*** |  |
|  | 15 | rs1370176 | rs1370176 | 0.731 | 0.687 **0.005546*** | **0.005546*** |  |
|  | 15 | rs1436953 | rs1436953 | 0.668 | 0.617 **0.002698*** | **0.002698*** |  |
|  | 15 | rs1436955 | rs1436955 | 0.786 | 0.749 **0.01309*** | **0.01309*** |  |
| *FTO* | 16 | rs8050136 | rs8050136 | 0.129 | 0.110 **0.08623**** | **0.08623**** |  |
| *TCF2 (HNF1B)* | 17 | rs757210 | rs757210 | 0.705 | 0.692 | 0.4470 |  |
|  |  | rs7501939 | rs7501939 | 0.723 | 0.704 | 0.2548 | **0.01** |
|  |  | rs4430796 | rs4430796 | 0.696 | 0.679 | 0.3066 |  |
| *SRR* | 17 | rs391300 | rs391300 | 0.718 | 0.713 | 0.7362 |  |
